# Anodal Transcranial Direct Current Stimulation Reduces Neuroinflammation and Cholinergic Dysregulation After Young Adult Traumatic Brain Injury

**DOI:** 10.3390/biom16101428

**Published:** 2026-10-01

**Authors:** Omer Faruk Anakok, Ali Osman Arslan, Sevdenur Akcay, Guven Akcay

**Affiliations:** 1 Department of Medical Biology, Faculty of Medicine, Bolu Abant İzzet Baysal University, 14030 Bolu, Turkey; omerfaruk.anakok@ibu.edu.tr (O.F.A.); aliosmanarslan@ibu.edu.tr (A.O.A.); 2 Department of Physiology, Faculty of Medicine, Bolu Abant İzzet Baysal University, 14030 Bolu, Turkey; sevdenuruzunn@gmail.com; 3 Department of Biophysics, Faculty of Medicine, Bolu Abant İzzet Baysal University, 14030 Bolu, Turkey

**Keywords:** traumatic brain injury, young adult brain injury, transcranial Direct Current Stimulation, tDCS polarity, neuroinflammation, cholinergic signaling, acetylcholinesterase, choline acetyltransferase, neuroplasticity

## Abstract

Traumatic brain injury (TBI) during childhood and adolescence can result in persistent motor and cognitive dysfunction due to secondary injury cascades involving neuroinflammation and disruption of neurotransmitter homeostasis. Although transcranial Direct Current Stimulation (tDCS) is a promising non-invasive neuromodulatory intervention, its effects on neuroinflammatory and cholinergic responses following young adult TBI remain incompletely understood. We investigated the effects of anodal and cathodal tDCS on functional outcomes, neuroinflammatory responses, and cholinergic regulatory markers following experimental young adult TBI. Two-month-old male Wistar rats underwent diffuse TBI using the Marmarou weight-drop model and were assigned to Sham, TBI, TBI + anodal tDCS, or TBI + cathodal tDCS groups. tDCS (1 mA, 30 min/day) was initiated 2 h post-injury and applied for 7 consecutive days. Functional outcomes were evaluated using the Open Field, Novel Object Recognition, and Y-maze tests. Neuroinflammatory responses were assessed by measuring tumor necrosis factor-alpha (TNF-α) and interleukin-1 beta (IL-1β) levels in the hippocampus and motor cortex using ELISA. Cholinergic regulatory changes were evaluated by analyzing acetylcholinesterase (AChE) and choline acetyltransferase (ChAT) protein expression using Western blotting. Young adult TBI induced significant locomotor and cognitive deficits, accompanied by increased TNF-α and IL-1β levels and alterations in AChE and ChAT expression in the hippocampus and motor cortex. Anodal tDCS significantly improved locomotor activity, recognition memory, and spatial working memory, while attenuating trauma-associated elevations in pro-inflammatory cytokines and reducing TBI-associated alterations in cholinergic regulatory markers. In contrast, cathodal tDCS produced limited behavioral and molecular effects. The effects of anodal stimulation were observed across both the hippocampus and motor cortex, suggesting coordinated modulation of neuroinflammatory and cholinergic responses. Collectively, these findings indicate that early anodal tDCS is associated with improved functional outcomes and modulation of neuroinflammatory and cholinergic responses following young adult TBI. These results support further investigation of anodal tDCS as a potential neuromodulatory approach for promoting recovery after TBI.

## 1. Introduction

Traumatic brain injury (TBI) remains one of the leading causes of morbidity and long-term neurological impairment in young adult populations. Sports-related collisions, falls, and impact injuries are particularly common among young individuals, and the developing brain may exhibit distinct vulnerability to secondary injury mechanisms following trauma [1,2]. Although the young adult brain possesses a high degree of plasticity, it may also remain susceptible to excitotoxicity, oxidative stress, neuroinflammation, and neurotransmitter imbalance. Therefore, identifying safe and effective neuromodulatory interventions for young adult TBI represents a critical clinical need.

Secondary injury cascades after TBI involve glutamate-mediated excitotoxicity, mitochondrial dysfunction, oxidative damage, and sustained neuroinflammatory responses [3]. In addition to excitotoxicity and oxidative stress, neuroinflammation represents a critical component of secondary injury following traumatic brain injury. Experimental and clinical studies have demonstrated that TBI induces rapid and sustained elevation of pro-inflammatory cytokines, including tumor necrosis factor-alpha (TNF-α) and interleukin-1 beta (IL-1β), particularly within the hippocampus and cortical regions [2,4]. These cytokines contribute to synaptic dysfunction, neuronal loss, and cognitive impairment by amplifying microglial activation and disrupting neurotransmitter balance. Importantly, inflammatory signaling has been shown to interact with cholinergic pathways, suggesting that post-traumatic neurotransmitter dysregulation may be partly driven by inflammatory cascades [4]. Therefore, modulation of neuroinflammatory mediators may represent a complementary mechanism underlying the therapeutic effects of neuromodulator interventions such as tDCS. In addition to glutamatergic dysregulation, substantial alterations occur within the cholinergic system, which plays a central role in attention, learning, memory, and motor integration. Experimental studies have demonstrated that TBI can disrupt cholinergic neurotransmission, although the direction of changes in cholinergic markers may vary depending on the injury model, severity, timing, and brain region examined. Several studies have reported increased acetylcholinesterase (AChE) activity together with reduced choline acetyltransferase (ChAT) expression following TBI, which may contribute to impaired cholinergic neurotransmission and cognitive deficits [5,6]. However, alterations in ChAT expression are not consistently reported across experimental TBI studies and increases in ChAT may also occur as a compensatory response to injury-related cholinergic dysfunction.

transcranial Direct Current Stimulation (tDCS) is a non-invasive neuromodulation technique capable of modulating cortical excitability in a polarity-dependent manner. Anodal stimulation generally enhances neuronal excitability, whereas cathodal stimulation reduces it [7]. Beyond acute excitability changes, accumulating evidence suggests that tDCS can influence neuroplasticity, synaptic signaling, oxidative balance, and inflamma-tory pathways [8].

Experimental studies have demonstrated that tDCS modulates glutamatergic trans-mission, calcium homeostasis, and inflammatory signaling in various neurological models. In our previous work, we showed that tDCS regulates glutamatergic pathways fol-lowing focal cerebral ischemia [7], improves motor recovery after mild traumatic brain injury [9], and alleviates motor and cognitive dysfunction in an experimental TBI model [1]. Moreover, tDCS has been reported to attenuate oxidative stress and neuroinflammation in neuropathic pain [10], epilepsy [11,12], and psychiatric disorder models [13]. Collectively, these findings suggest that tDCS may exert multifaceted neuroprotective effects by modulating key secondary injury mechanisms.

However, the impact of tDCS on cholinergic signaling following traumatic brain injury remains insufficiently characterized. In particular, studies investigating the effects of anodal and cathodal tDCS on AChE and ChAT expression in young adult TBI models are limited. Given the essential role of cholinergic neurotransmission in cognitive performance and motor coordination, targeting this pathway may represent a meaningful therapeutic strategy in young adult TBI.

Accordingly, we hypothesized that the therapeutic effects of tDCS following young adult TBI are polarity-dependent and that anodal stimulation would improve functional recovery by reducing trauma-associated neuroinflammatory responses and cholinergic dysregulation. Therefore, this study investigated the differential effects of anodal and cathodal tDCS on behavioral outcomes, TNF-α and IL-1β levels, and AChE and ChAT protein expression in the hippocampus and motor cortex following experimental young adult TBI. Using a diffuse TBI model induced by the Marmarou weight-drop method in two-month-old male Wistar rats, we evaluated the effects of early tDCS administered at 1 mA for 30 min/day for seven consecutive days, beginning 2 h after injury. Functional recovery was assessed using the Open Field, Novel Object Recognition, and Y-maze tests, while neuroinflammatory and cholinergic changes were evaluated in the hippocampus and motor cortex.

## 2. Materials and Methods

### 2.1. Animals and Experimental Design

The study was conducted in accordance with the European Community Directive (86/609/EEC) for the care and use of laboratory animals and was approved by the Animal Experiments Local Ethics Committee (Approval No: 82678388). All experimental procedures complied with institutional and international guidelines for animal welfare and experimental research.

Two-month-old male Wistar albino rats (200–230 g) were used in this study. This age was selected because it corresponds to a young adult developmental stage, which is considered relevant for modeling young adult traumatic brain injury. Only male animals were included to minimize potential variability associated with hormonal fluctuations and sex-dependent neurobiological responses. Animals were obtained from the Experimental Animal Research Center and housed under standard laboratory conditions (23 ± 2 °C, relative humidity 50 ± 5%, 12-h light/dark cycle) with ad libitum access to standard pellet chow and water. Prior to the experimental procedures, all animals were acclimatized to the laboratory environment for one week.

Animals were monitored daily throughout the study, and all efforts were made to minimize pain and distress. Sample size was determined based on previous experimental studies using similar traumatic brain injury models [1,8]. Animals were randomly assigned to experimental groups. No animals were excluded from study, and no mortality or experimental loss occurred during the experimental period. All animals were included in the final statistical analysis.

Following completion of the experimental procedures and behavioral assessments, animals were euthanized under deep anesthesia, and brain tissues were rapidly collected for subsequent molecular, and biochemical analyses.

### 2.2. Experimental Groups

Animals were randomly assigned to four groups:

(1) Control (Sham): Animals underwent anesthesia and surgical preparation without weight impact and did not receive active stimulation.
(2) TBI: Animals were subjected to the Marmarou weight-drop injury and underwent the same procedures without receiving tDCS treatment.
(3) Anodal tDCS + TBI: Animals received TBI followed by anodal tDCS (1 mA, 30 min/day) initiated 2 h post-injury and continued for 7 consecutive days.
(4) Cathodal tDCS + TBI: Animals received TBI followed by cathodal tDCS (1 mA, 30 min/day) initiated 2 h post-injury and continued for 7 consecutive days.

**Figure 1:**
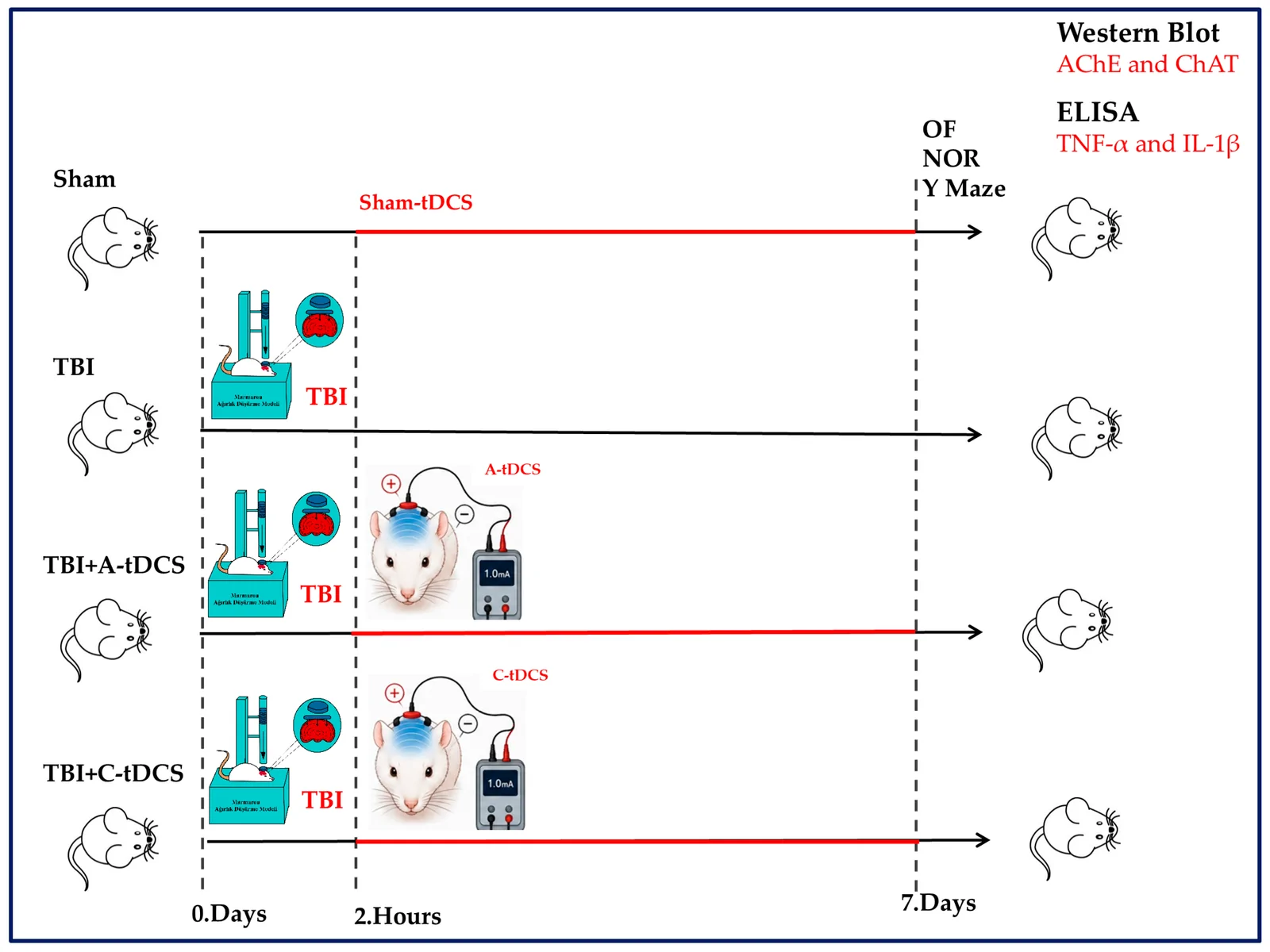
Experimental timeline and study design. Schematic representation of the experimental protocol. Rats were divided into four groups: Sham, TBI, TBI + Cathodal tDCS (C-tDCS), and TBI + Anodal tDCS (A-tDCS). Traumatic brain injury was induced on Day 0 using the Marmarou weight-drop model. transcranial Direct Current Stimulation (1 mA, 30 min/day) was initiated 2 h after TBI and continued for 7 consecutive days. Behavioral assessments, including Open Field (OF), Novel Object Recognition (NOR), and Y-maze tests, were performed after completion of the stimulation period. Following behavioral evaluation, hippocampal and motor cortex tissues were collected for Western blot analysis of acetylcholinesterase (AChE) and choline acetyltransferase (ChAT) expression.

Behavioral and molecular assessments were performed by investigators blinded to group allocation. A schematic overview of the experimental design and study timeline is presented in Figure 1. The sample size (*n* = 8 per group) was determined based on group sizes used in our previous experimental TBI and tDCS studies employing comparable animal models and outcome measures [7,8].

### 2.3. Traumatic Brain Injury Model

Traumatic brain injury was induced using a modified Marmarou weight-drop model [9]. Rats were anesthetized with 5% isoflurane in oxygen for induction and maintained with 2% isoflurane during the procedure. A stainless-steel disc (10 mm diameter, 3 mm thickness) was placed on the intact skull between bregma and lambda and affixed using dental acrylic to distribute the impact force. No craniotomy, craniectomy, or scalp incision was performed. A 250-g brass weight was dropped from a height of 1 m through a vertical guide tube onto the steel disc, producing diffuse brain injury [4].

Sham animals underwent the identical experimental preparation, including the same isoflurane anesthesia protocol, positioning, placement and fixation of the stainless-steel disc using dental acrylic, and handling procedures, but did not receive the weight-drop impact. Thus, the only procedural difference between the Sham and TBI groups was the absence of the traumatic weight-drop impact in the Sham group. Following the procedure, animals were returned to their home cages and monitored until full recovery from anesthesia [8].

### 2.4. Transcranial Direct Current Stimulation (tDCS)

transcranial Direct Current Stimulation was initiated 2 h after TBI induction. Stimulation was delivered once daily for 7 consecutive days [7]. During each tDCS session, animals were maintained under isoflurane anesthesia to prevent movement and ensure consistent electrode positioning. A constant-current stimulator was used to apply 1 mA direct current for 30 min per session with a maximum current intensity of ±1000 μA and a current resolution of 0.01 mA, as previously described [8]. The active electrode was positioned over the motor cortex region, while the reference electrode was placed over the contralateral supraorbital area. The stimulation electrodes were circular disc electrodes with an external diameter of 1.2 cm, corresponding to an electrode area of approximately 1.13 cm^2^. At the applied current of 1 mA, the calculated current density was approximately 0.88 mA/cm^2^. Electrode-skin contact was ensured using saline-soaked sponge pads. Animals in the anodal tDCS group received anodal stimulation over the motor cortex, whereas animals in the cathodal group received cathodal stimulation. Control and TBI groups underwent identical handling procedures, including isoflurane anesthesia and electrode placement, but without current delivery (sham stimulation). Before each stimulation session, electrode contact and impedance were checked using the impedance-monitoring function of the stimulator, and the electrode position and contact were adjusted as necessary to ensure adequate electrical coupling before current delivery was initiated.

### 2.5. Behavioral Assessments

Behavioral tests were performed after completion of the 7-day stimulation protocol by observers blinded to the experimental group allocation. All behavioral tests were conducted on the same day in a standardized sequence. The Open Field test was performed first, followed by the Novel Object Recognition (NOR) test, and the Y-maze test was conducted in the afternoon. This testing sequence was kept consistent across all experimental groups.

#### 2.5.1. Open Field Test (OF)

Locomotor activity was evaluated in a black plexiglass arena (80 × 80 × 40 cm) divided into 16 equal squares. Each rat was placed in the center of the arena and allowed to explore freely for 5 min. Total distance (cm), velocity (cm/s) and frequency of entrance (frequency) were recorded using a video tracking system [7,14]. 

#### 2.5.2. Novel Object Recognition (NOR) Test

Short-term recognition memory was assessed using the NOR test, consisting of habituation, training, and retention phases. During the retention phase, one familiar object was replaced with a novel object, and the animals were allowed to explore the arena freely for 5 min. The time spent exploring the novel and familiar objects was recorded using a video tracking system. The percentage of exploration time directed toward the novel object was calculated as the time spent exploring the novel object divided by the total object exploration time (novel object + familiar object) and multiplied by 100 [7]. The percentage of novel-object exploration time was used as the primary measure of recognition memory.

#### 2.5.3. Y-Maze Test

Spatial working memory was assessed using a Y-maze with three identical arms (50 cm length, 20 cm height, 10 cm width, 120° angle). Each rat was placed in the center of the maze and allowed to explore all three arms freely for 5 min. Arm entries were recorded using a video tracking system. Spontaneous alternation percentage was calculated as the number of sequential triads containing entries into all three arms without repetition, divided by the total number of possible alternations (total arm entries − 2), multiplied by 100. The spontaneous alternation percentage was used as the primary measure of spatial working memory [7].

### 2.6. Tissue Collection and Molecular Analyses

Following completion of behavioral testing, all animals were euthanized under deep anesthesia. The brains were rapidly removed, and the hippocampus and motor cortex were dissected on ice. The motor cortical tissue was identified based on established neuroanatomical landmarks and the anatomical organization of the rat frontal cortex. The dissected motor cortex samples included the anatomically defined primary motor cortex (M1) and secondary motor cortex (M2) regions.

Each experimental group consisted of eight animals (*n* = 8). For each experimental group, hippocampal and motor cortex tissues from five animals were allocated for ELISA analyses (*n* = 5), whereas tissues from the remaining three animals were allocated for Western blot analyses (*n* = 3). Thus, the eight animals in each experimental group were distributed between the two biochemical analyses according to a predefined tissue allocation scheme. Tissues were snap-frozen in liquid nitrogen and stored at −80 °C until analysis.

#### 2.6.1. ELISA Analysis

Hippocampal and motor cortex tissues obtained from five animals per experimental group were homogenized in phosphate-buffered saline (PBS, pH 7.4) and centrifuged at 12,000 rpm for 20 min at 4 °C. The supernatants were collected for analysis. TNF-α and IL-1β levels were quantified using commercially available rat-specific ELISA kits (TNF-α ELISA Kit, Cat. No. RE1060RG; IL-1β ELISA Kit, Cat. No. RE1074RG; ReedBiotech Ltd., Wuhan, China.) according to the manufacturer’s instructions. Before ELISA measurements, the total protein concentration of each tissue homogenate was determined using the Bradford assay (Bio-Rad Laboratories, Hercules, CA, USA). Samples were normalized to an equal protein concentration of 1 mg/mL using phosphate buffer (0.1 M, pH 7.4). Absorbance was measured at 450 nm, and cytokine concentrations were calculated from standard curves. All measurements were performed in duplicate.

#### 2.6.2. Western Blot Analysis

Protein expression levels of acetylcholinesterase (AChE) and choline acetyltransferase (ChAT) were determined by Western blot analysis. Following tissue dissection, hippocampal and motor cortex tissues from three animals per experimental group were used for Western blot analysis. The motor cortex samples included the primary (M1) and secondary (M2) motor cortical areas. Tissues were weighed and homogenized by ultrasonication in 800 µL of Triton lysis buffer containing 1% Triton X-100, 20 mM Tris-HCl (pH 7.4), 150 mM NaCl, 5 mM EGTA, 10 mM EDTA, 10% glycerol, 20 mM HEPES, and protease inhibitor cocktail (Roche, Basel, Switzerland). Sonication was performed using a sonic dismembrator with a microtip probe at a power output of 40 W for three cycles of 5 s each, with 15-s intervals between sonications, while samples were maintained in an ice bath. The homogenates were then centrifuged at 20,000× *g* for 30 min at 4 °C, and the supernatants were collected and stored at −80 °C until analysis.

Protein concentrations were determined using the Bradford assay. Equal amounts of protein (15 µg per sample) were separated on 7.5% polyacrylamide gels. The separated proteins were transferred onto PVDF membranes using electroblotting with a Mini-PROTEAN Tetra Cell system (Bio-Rad Laboratories, Hercules, CA, USA). Membranes were blocked with 5% non-fat dried milk in TBST buffer (20 mM Tris-HCl, 0.5 M NaCl, and 0.05% Tween-20, pH 7.6) for 1 h at room temperature and subsequently incubated for 2 h at room temperature with primary antibodies against ChAT (Abcam, Cambridge, UK; Cat. No. [AB223346]) and AChE (Abcam, Cambridge, UK; Cat. No. [AB31276]) at a dilution of 1:1000. Following washing with TBS-T, membranes were incubated for 1 h at room temperature with alkaline phosphatase-conjugated secondary antibodies (Cat. No. [ab6721], [Abcam, Cambridge, UK]) at a dilution of 1:3000 in 5% non-fat dried milk in TBS-T. β-Actin was used as the loading control, and AChE and ChAT protein expression levels were normalized to the corresponding β-actin signal. Immunoreactive protein bands were visualized using an AP-conjugated substrate kit (Bio-Rad Laboratories, Hercules, CA, USA) and captured using an InfinityCapt imaging system (Vilber Lourmat, Marne-la-Vallée, France). Band intensities were quantified by densitometric analysis using ImageJ software v1.53q (National Institutes of Health, Bethesda, MD, USA).

### 2.7. Statistical Analysis

Statistical analyses were performed using IBM SPSS Statistics version 20.0 (IBM Corp., Armonk, NY, USA). Data are presented as mean ± standard error of the mean (SEM). The normality of data distribution was assessed using the Shapiro–Wilk test, while the homogeneity of variances was evaluated using Levene’s test. For datasets meeting the assumptions of normality and homogeneity of variance, intergroup comparisons were performed using one-way analysis of variance (ANOVA), followed by Tukey’s multiple-comparisons post hoc test. For datasets that did not satisfy these assumptions, the Kruskal–Wallis test was applied, followed by Dunn’s post hoc test with Bonferroni adjustment for multiple comparisons when the Kruskal–Wallis test was significant. The Bonferroni adjustment was used to control the family-wise type I error associated with multiple pairwise comparisons. Outliers were evaluated during data analysis; however, no experimental data were excluded from the final analysis. Exact *p*-values were reported where appropriate, and statistical significance was defined as *p* < 0.05.

## 3. Results

### 3.1. tDCS Differentially Modulates Locomotor and Cognitive Recovery After Young Adult TBI

**Figure 2:**
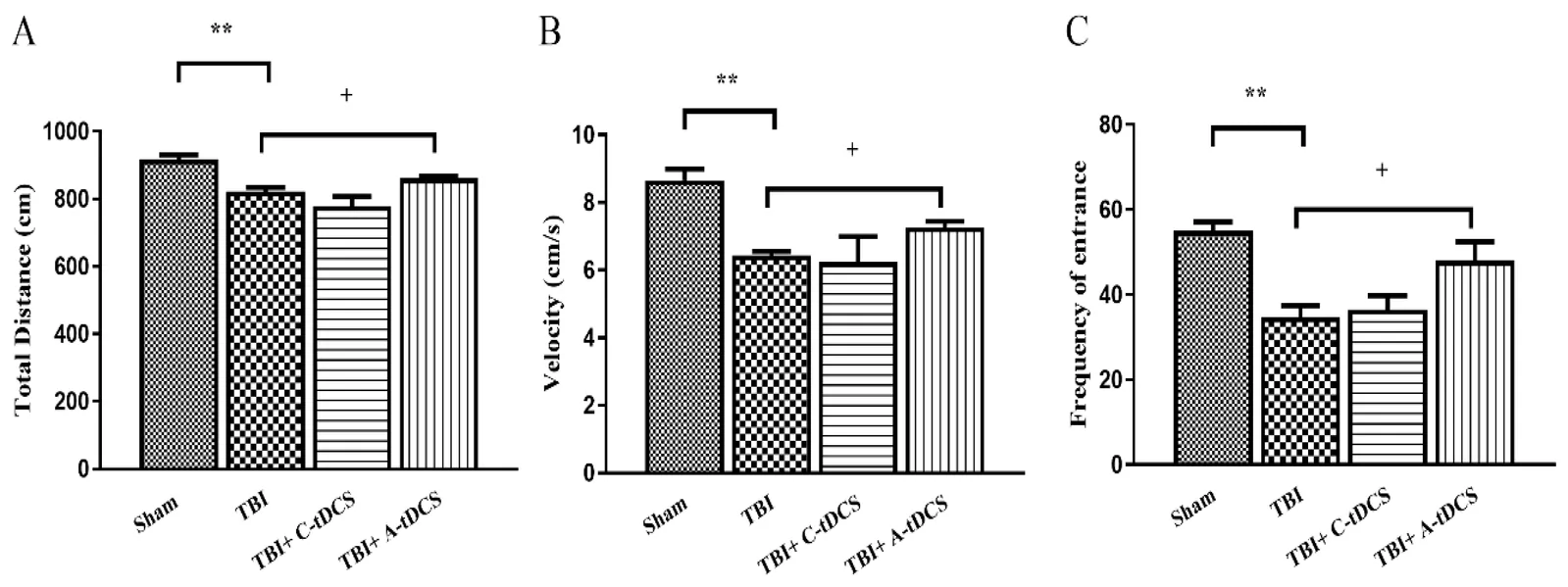
Effects of tDCS on locomotor activity in the Open Field test. (**A**) Total distance (cm), (**B**) velocity (cm/s), and (**C**) frequency of entrance in Sham, TBI, TBI + Cathodal tDCS (C-tDCS), and TBI + Anodal tDCS (A-tDCS) groups. TBI significantly reduced locomotor parameters compared to the Sham group. Anodal tDCS significantly improved total distance, velocity, and frequency of entrance compared to the TBI group, whereas cathodal stimulation produced limited effects. Data are presented as mean ± SEM (*n* = 8 per group). ** *p* < 0.01 vs. Sham; + *p* < 0.05 vs. TBI.

TBI significantly reduced total distance compared to the Sham group (** *p* < 0.01) (Figure 2A). Cathodal tDCS did not significantly improve total distance compared to TBI alone. In contrast, anodal tDCS significantly increased total distance compared to the TBI group (+ *p* < 0.05), partially improving locomotor activity toward sham levels. A significant decrease in velocity was observed in the TBI group compared to Sham (** *p* < 0.01). Anodal tDCS significantly improved velocity compared to TBI (+ *p* < 0.05), whereas cathodal stimulation did not produce a significant change (Figure 2B). TBI significantly reduced the frequency of entrance compared to Sham (** *p* < 0.01) (Figure 2C). Anodal tDCS significantly increased entrance frequency compared to TBI (+ *p* < 0.05), while cathodal tDCS showed no significant improvement. Overall, anodal tDCS markedly ameliorated TBI-induced locomotor impairment, whereas cathodal stimulation showed limited behavioral benefit.

**Figure 3:**
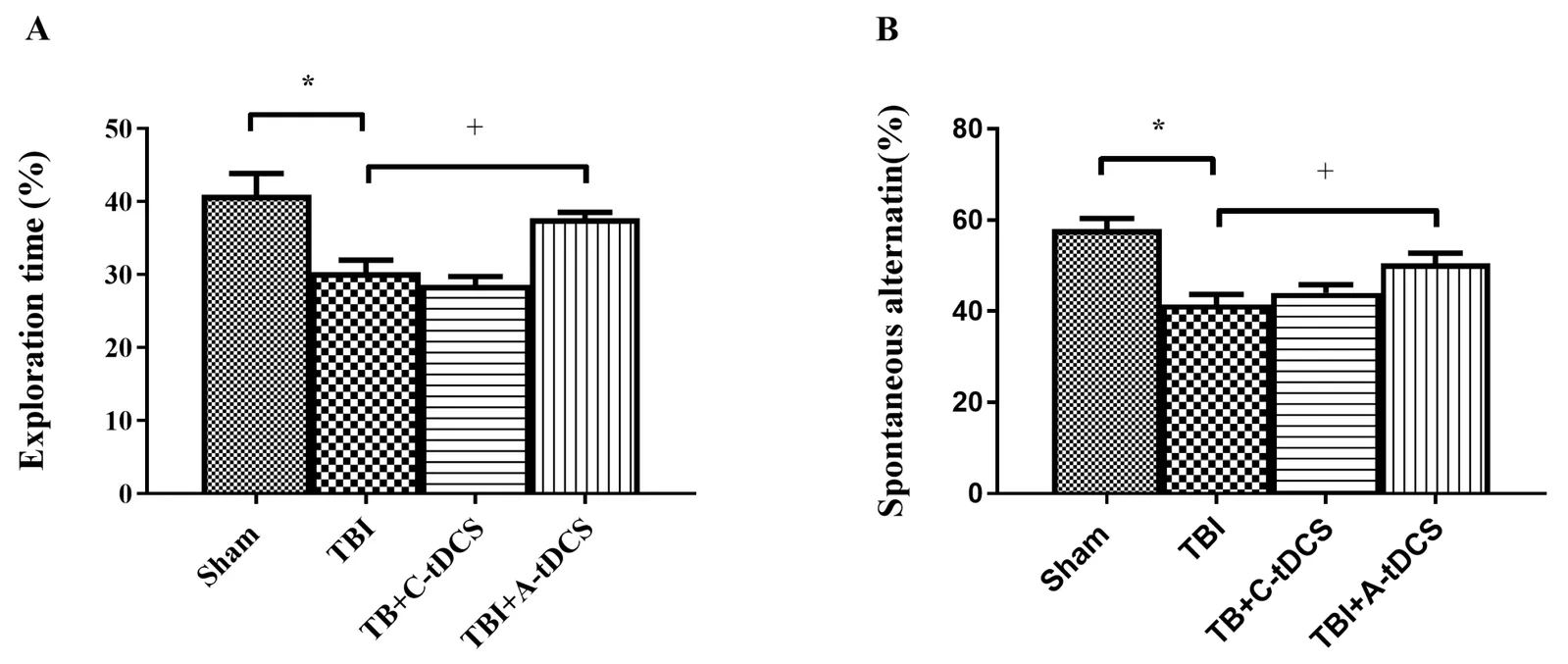
Effects of tDCS on cognitive performance. (**A**) Exploration time (%) in the Novel Object Recognition (NOR) test and (**B**) spontaneous alternation (%) in the Y-maze test in Sham, TBI, TBI + Cathodal tDCS (C-tDCS), and TBI + Anodal tDCS (A-tDCS) groups. TBI significantly reduced novel object exploration and alternation performance compared to the Sham group. Anodal tDCS significantly improved both parameters relative to the TBI group, whereas cathodal stimulation showed no significant improvement. Data are presented as mean ± SEM (*n* = 8 per group). * *p* < 0.05 vs. Sham; + *p* < 0.05 vs. TBI.

TBI significantly decreased the percentage of exploration time directed toward the novel object compared with the Sham group (*p* < 0.05), indicating impaired recognition memory (Figure 3A). Anodal tDCS significantly increased novel-object exploration time (%) compared with the TBI group (+ *p* < 0.05), indicating improved recognition memory performance. Cathodal tDCS did not significantly improve novel-object exploration performance.

Spontaneous alternation percentage was significantly reduced in the TBI group compared with the Sham group (*p* < 0.05) (Figure 3B). Anodal tDCS significantly improved spontaneous alternation compared with the TBI group (+ *p* < 0.05), suggesting improved spatial working memory. Cathodal stimulation did not significantly alter spontaneous alternation performance.

### 3.2. Anodal tDCS Attenuates TBI-Induced Neuroinflammatory Responses in the Hippocampus and Motor Cortex

**Figure 4:**
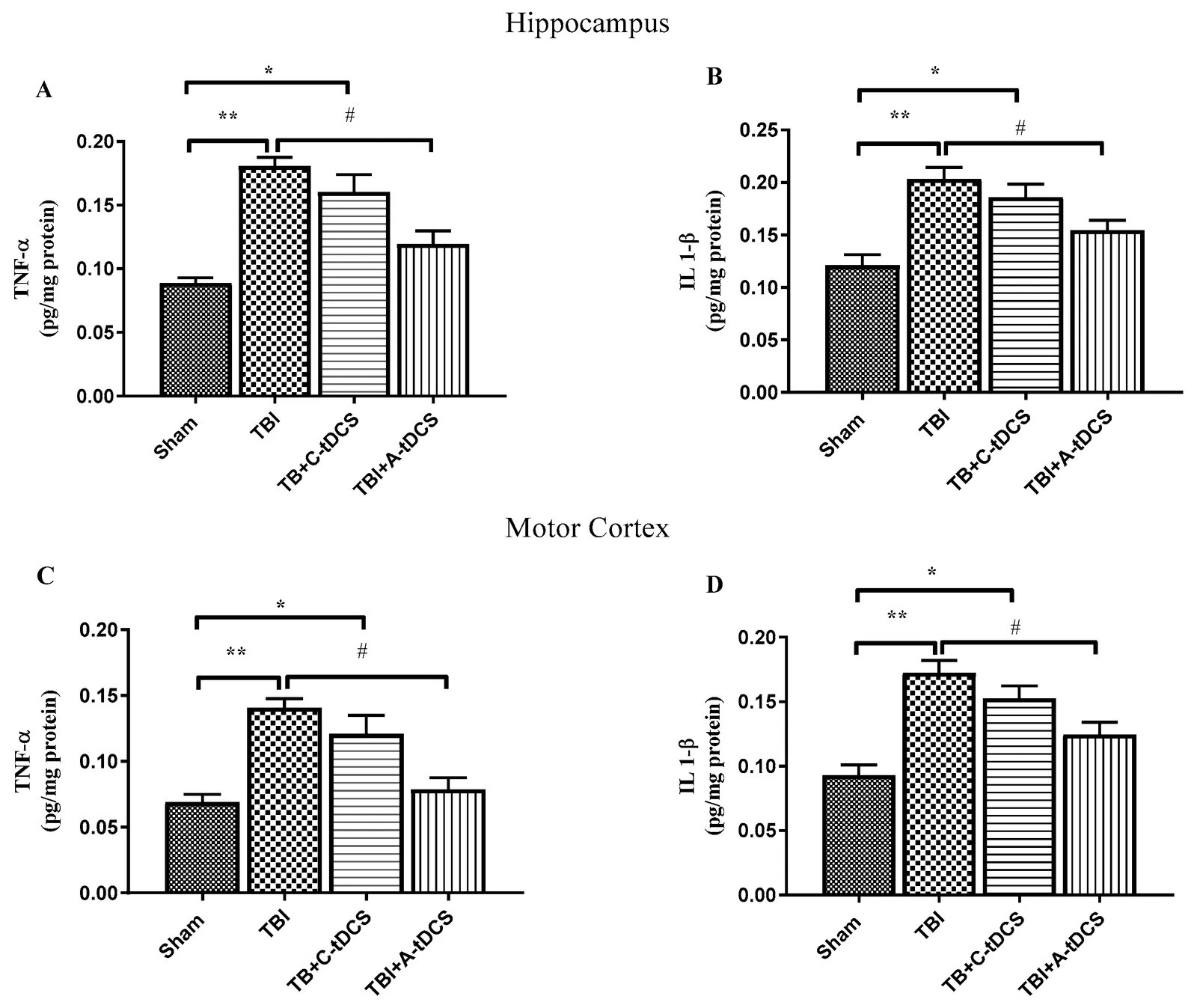
Effects of tDCS on pro-inflammatory cytokine levels in the hippocampus and motor cortex. (**A**) TNF-α and (**B**) IL-1β levels in the hippocampus; (**C**) TNF-α and (**D**) IL-1β levels in the motor cortex of Sham, TBI, TBI + Cathodal tDCS (C-tDCS), and TBI + Anodal tDCS (A-tDCS) groups. TBI significantly increased TNF-α and IL-1β levels in both brain regions compared to the Sham group. Anodal tDCS significantly reduced trauma-induced elevations in pro-inflammatory cytokines relative to the TBI group, whereas cathodal stimulation produced a partial or less pronounced reduction. Data are presented as mean ± SEM (*n* = 5 per group). * *p* < 0.05, ** *p* < 0.01 vs. Sham; # *p* < 0.05 vs. TBI.

Traumatic brain injury significantly increased pro-inflammatory cytokine levels in both the hippocampus and motor cortex compared to the Sham group. In the hippocampus, TNF-α levels were markedly elevated in the TBI group (** *p* < 0.01 vs. Sham) (Figure 4A). Cathodal tDCS produced a partial reduction in TNF-α; however, this decrease was not sufficient to fully normalize levels. In contrast, anodal tDCS significantly reduced hippocampal TNF-α compared to the TBI group (*p* < 0.05), bringing values closer to those observed in Sham animals.

Similarly, hippocampal IL-1β levels were significantly increased following TBI (** *p* < 0.01 vs. Sham) (Figure 4B). Anodal tDCS significantly attenuated this elevation (# *p* < 0.05 vs. TBI), whereas cathodal stimulation resulted in a modest but less pronounced reduction.

In the motor cortex, TBI induced a significant increase in TNF-α levels compared to Sham (** *p* < 0.01) (Figure 4C). Anodal tDCS significantly decreased TNF-α expression relative to the TBI group (# *p* < 0.05), while cathodal stimulation showed only partial improvement. A similar pattern was observed for IL-1β in the motor cortex (Figure 4D), where TBI significantly elevated cytokine levels (** *p* < 0.01 vs. Sham), and anodal tDCS significantly reduced these levels compared to TBI (# *p* < 0.05). Cathodal stimulation did not produce a statistically significant normalization.

Overall, these findings indicate that young adult TBI induces a robust neuroinflammatory response in both hippocampal and cortical regions, and that early anodal tDCS markedly attenuates trauma-induced elevations in TNF-α and IL-1β, whereas cathodal stimulation exerts limited anti-inflammatory effects.

### 3.3. Anodal tDCS Attenuates Trauma-Induced Cholinergic Dysregulation in the Hippocampus and Motor Cortex

**Figure 5:**
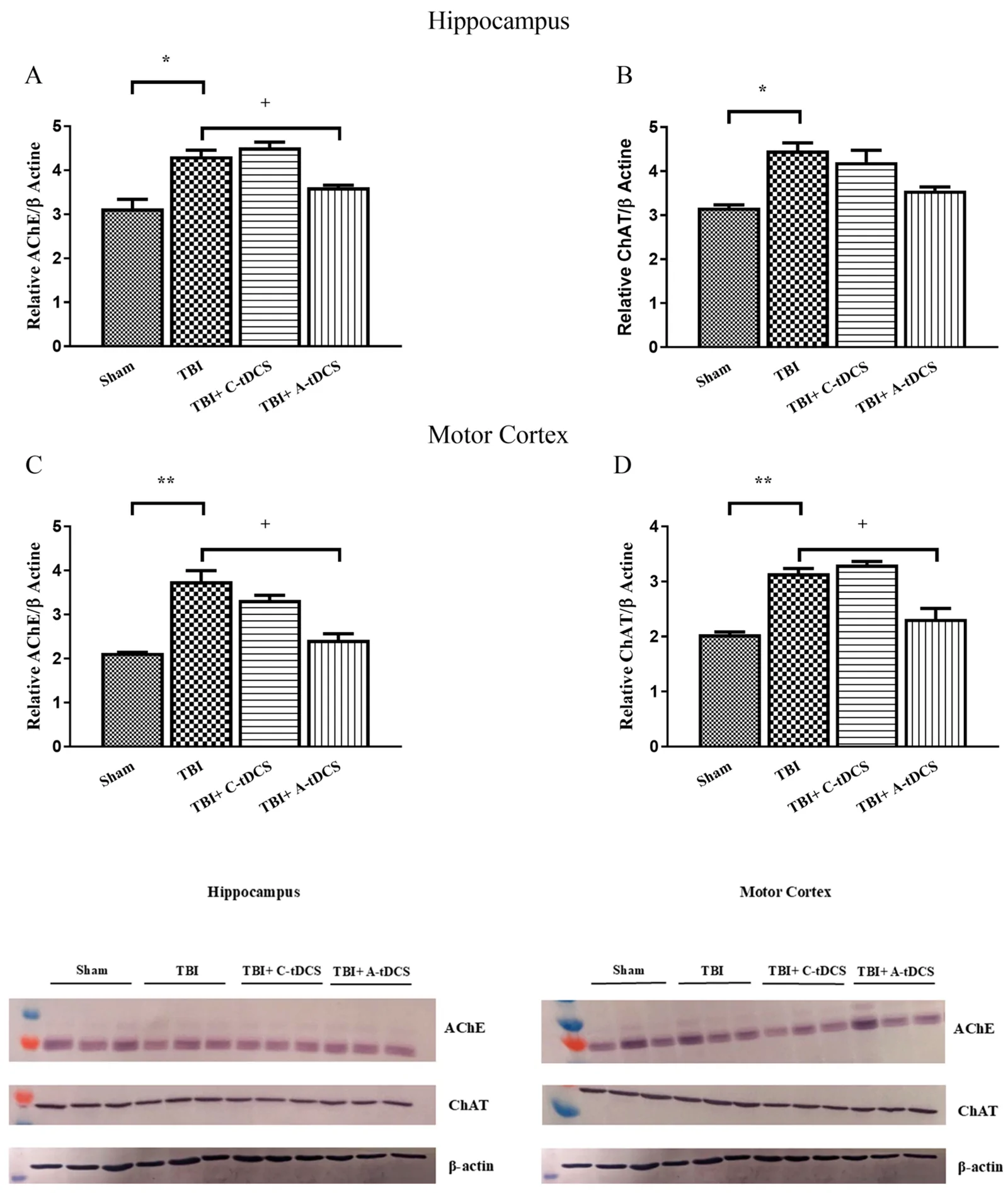
Effects of tDCS on cholinergic protein expression in the hippocampus and motor cortex. Representative Western blot images and corresponding densitometric analyses of acetylcholinesterase (AChE) and choline acetyltransferase (ChAT) expression in the hippocampus and motor cortex of Sham, TBI, TBI + Cathodal tDCS (C-tDCS), and TBI + Anodal tDCS (A-tDCS) groups. (**A**) Representative immunoblots and relative AChE/β-actin expression in the hippocampus; (**B**) representative immunoblots and relative ChAT/β-actin expression in the hippocampus; (**C**) representative immunoblots and relative AChE/β-actin expression in the motor cortex; and (**D**) representative immunoblots and relative ChAT/β-actin expression in the motor cortex. β-actin was used as the loading control. Representative immunoblots from three animals per group are shown, and densitometric analyses were performed using samples from the three animals allocated to Western blot analysis in each group (*n* = 3). Data are presented as mean ± SEM. Molecular weight markers are indicated in kDa. * *p* < 0.05, ** *p* < 0.01 vs. Sham; + *p* < 0.05 vs. TBI. Original Western blot images can be found in Supplementary Figures S1–S5.

Relative AChE/β-actin expression was significantly increased in the TBI group compared with the Sham group (*p* < 0.05) (Figure 5A). Anodal tDCS significantly reduced AChE expression compared with the TBI group (+ *p* < 0.05), whereas cathodal tDCS did not produce a significant reduction.

ChAT expression was significantly elevated in the TBI group compared with the Sham group (*p* < 0.05) (Figure 5B). Both anodal and cathodal tDCS showed a reduction in ChAT expression compared with the TBI group, with the reduction being more pronounced following anodal stimulation; however, neither reduction reached statistical significance.

TBI significantly increased AChE expression in the motor cortex compared with the Sham group (*p* < 0.01) (Figure 5C). Anodal tDCS significantly decreased AChE levels relative to the TBI group (+ *p* < 0.05), whereas cathodal stimulation resulted in a mild but non-significant reduction. ChAT expression was also significantly increased in the TBI group compared with the Sham group (*p* < 0.01) (Figure 5D). Anodal tDCS significantly reduced ChAT expression compared with the TBI group (+ *p* < 0.05), whereas cathodal stimulation did not produce a significant change.

## 4. Discussion

In the present study, we demonstrate that anodal and cathodal tDCS differentially affect behavioral recovery, neuroinflammation, and cholinergic signaling following traumatic brain injury (TBI) in young adult rats. Our findings indicate that TBI induces locomotor and cognitive impairments accompanied by robust increases in TNF-α and IL-1β levels and dysregulation of cholinergic markers in both the hippocampus and motor cortex. Importantly, early anodal tDCS improved behavioral outcomes and significantly attenuated several trauma-associated molecular alterations, particularly inflammatory cytokines and selected cholinergic markers, whereas cathodal stimulation exerted limited effects. These results suggest that excitability-enhancing neuromodulation may attenuate secondary injury cascades at both inflammatory and neurotransmitter levels.

Neuroinflammation represents a central component of secondary injury following TBI and is increasingly viewed as a driver of chronic neurodegenerative processes after injury [14]. Rapid activation of microglia and astrocytes leads to sustained release of pro-inflammatory cytokines such as TNF-α and IL-1β, which amplify neuronal dysfunction and impair synaptic plasticity [15,16], and prolonged inflammatory signaling can further exacerbate tissue damage and functional decline [17]. Microglial responses are also dynamic, with early potentially reparative effects but persistent pro-inflammatory polarization contributing to chronic cognitive impairment, which may help explain why early intervention is biologically relevant in TBI [17,18]. Elevated cytokine signaling disrupts long-term potentiation, alters glutamatergic transmission, and contributes to memory deficits. In the present study, TBI significantly increased TNF-α and IL-1β levels in both examined brain regions, confirming activation of inflammatory pathways following diffuse TBI in young adult rats. Notably, anodal tDCS markedly reduced cytokine elevations. This anti-inflammatory effect may be associated with modulation of neuroinflammatory processes, potentially involving changes in microglial activation and downstream inflammatory signaling pathways, as suggested by previous neuromodulation studies [11]. Related preclinical evidence suggests that suppression of microglial and astrocytic activation, including TLR4/NF-κB-associated signaling, may reduce IL-1β and TNF-α expression and improve neurological outcomes after TBI [19]. Similarly, previous studies have reported that modulation of IFN-β signaling can attenuate post-traumatic neuroinflammation, reduce long-term microglial activation, and improve motor and cognitive recovery [20]. These findings provide potential mechanistic context for the anti-inflammatory effects observed in the present study. However, microglial or astrocytic activation, TLR4/NF-κB signaling, and IFN-β-related pathways were not directly assessed in our experiments; therefore, their involvement in the effects of anodal tDCS remains speculative. Accordingly, the observed reduction in inflammatory markers may be consistent with attenuation of neuroinflammatory processes, but the specific cellular and molecular pathways underlying this effect cannot be determined from the present data.

The interaction between neuroinflammation and cholinergic signaling provides an additional mechanistic layer. The cholinergic system plays a crucial role in cognitive processing, particularly in hippocampal-dependent memory and cortical attention networks [6]. Basal forebrain–cortical cholinergic projections are especially important for attentional performance and are vulnerable to TBI-related disruption [21]. Following TBI, alterations in AChE and ChAT expression may reflect disrupted acetylcholine turnover and compensatory responses to synaptic imbalance [5]. Recent work further supports that cholinergic disruptions underlie persistent attentional deficits after TBI and that chronic downregulation of ChAT can impair acetylcholine bioavailability [21]. In our study, TBI increased both AChE and ChAT expression, indicating alterations in cholinergic marker expression following trauma. Although several previous studies have reported reduced ChAT expression following TBI [5,6], the increase observed in the present study may reflect differences in injury severity experimental model, brain region, or time point examined. This interpretation is consistent with evidence that cholinergic markers after TBI can show bidirectional and time-dependent changes, with some studies reporting acute increases rather than only chronic reductions. Increased ChAT expression may also represent a compensatory response aimed at maintaining acetylcholine synthesis in the setting of trauma-induced cholinergic dysfunction. Thus, increased ChAT expression should not necessarily be interpreted as evidence of enhanced cholinergic neurotransmission, particularly in the presence of increased AChE expression. This distinction is important because enzyme expression, soluble cholinergic markers, and effective neurotransmission do not always change in parallel [22].

Importantly, inflammatory cytokines are known to interfere with cholinergic neurotransmission. TNF-α and IL-1β can impair synaptic receptor trafficking, reduce neuro-transmitter release efficiency, and alter enzyme expression patterns. Conversely, the cho-linergic anti-inflammatory pathway, mediated via α7 nicotinic acetylcholine receptors, modulates cytokine production [23]. This bidirectional relationship is consistent with work showing that cholinergic-targeted interventions after TBI can alter acetylcholine signaling without necessarily restoring behavior, underscoring the complexity of linking marker normalization to functional recovery [21]. Therefore, the observed changes in AChE and ChAT expression following anodal tDCS may be associated with reduced inflammatory signaling. These findings raise the possibility that anodal stimulation may concurrently modulate cytokine-associated synaptic disruption and cholinergic signaling, potentially contributing to the observed behavioral improvements. Direct support for this interpretation comes from newer preclinical data showing that TBI impairs task-evoked acetylcholine signaling in the medial prefrontal cortex and is associated with structural abnormalities in cholinergic neurons linked to attentional dysfunction [24].

The polarity-dependent nature of these effects is consistent with established tDCS physiology. Anodal stimulation increases cortical excitability and promotes activity-dependent synaptic plasticity through NMDA receptor-dependent mechanisms [7,25]. tDCS-induced plasticity is also calcium-dependent and involves glutamatergic signaling, with reduced GABAergic activity proposed as a gating mechanism for these after-effects [12]. Enhanced cortical excitability induced by anodal tDCS may strengthen residual neural circuits and facilitate adaptive network reorganization during recovery [26]. Experimental work further indicates that anodal stimulation can enhance LTP formation and increase BDNF expression in hippocampal and prefrontal regions, supporting a plausible biological basis for improved recovery-related plasticity [7,8,26]. However, the observed functional improvements may also reflect neuroprotective effects of early tDCS, including attenuation of secondary injury processes and preservation of neuronal integrity. Thus, neuroprotection and neuroplasticity may represent complementary mechanisms contributing to the functional benefits observed in the present study. Because neuronal survival and direct measures of neuroplasticity were not assessed, the relative contribution of these mechanisms cannot be determined. Conversely, cathodal stimulation generally decreases cortical excitability and, when applied to already functionally depressed regions after acute TBI, may further reduce neuronal activity, potentially limiting its therapeutic efficacy. Therefore, the polarity of stimulation appears to be a critical determinant of whether neuromodulation promotes or constrains recovery-related neuroplasticity [27].

The young adult model used in this study provides relevant translational insights into the effects of early neuromodulation following TBI. Young adult brains retain substantial neuroplastic capacity while remaining susceptible to inflammatory and excitotoxic cascades [28]. Early intervention targeting secondary injury mechanisms may therefore be particularly relevant for functional recovery following TBI, especially because inflammatory mediators can exert stage-dependent effects and may be more amenable to modulation early after injury [17]. Our findings suggest that timely excitability enhancing neuromodulation can shift the post-traumatic molecular environment from a pro-inflammatory, dysregulated state toward a more permissive condition for functional recovery. This interpretation is broadly consistent with preclinical TBI literature showing that tDCS can mitigate deficits in motor coordination, working memory, spatial memory, and impulsivity, while also increasing neuronal activity and focal plasticity-related markers in stimulated cortex [29]. Notably, initiation of stimulation within 2 h after injury models a clinically feasible early intervention window, suggesting that timely neuromodulation may interfere with evolving secondary injury cascades before irreversible network deterioration occurs [30,31].

Several limitations warrant consideration. We did not directly assess acetylcholine levels, cholinergic enzyme activity, receptor function, or long-term outcomes. In addition, only male animals were included, limiting the generalizability of the findings and precluding assessment of sex-dependent responses to TBI and tDCS. The Western blot analyses were performed using three biological replicates per group (*n* = 3), which limits the statistical power and precision of these molecular findings. Therefore, the Western blot results should be considered exploratory and interpreted with appropriate caution. Although β-actin was used for Western blot normalization, its stability under TBI conditions was not independently validated; therefore, TBI-related alterations in β-actin expression cannot be completely excluded. Future studies incorporating alternative loading controls, electrophysiological and microglial assessments, direct measures of cholinergic neurotransmission, and longer-term behavioral follow-up may further clarify the mechanisms underlying the observed effects. The concurrent changes in inflammatory and cholinergic markers may indicate that tDCS influences multiple post-traumatic processes; however, these associations do not establish a causal or systems-level mechanism [32]. This multimodal approach is consistent with broader tDCS literature emphasizing the value of combining behavioral outcomes with neurophysiological and imaging-based measures to better characterize stimulation effects in TBI [33,34]. Finally, behavioral performance was assessed only after the 7-day intervention, without pre-injury baseline testing. Although animals were matched for age, sex, and body weight and maintained under standardized conditions, the absence of individual baseline measurements prevents assessment of within-animal behavioral change. Thus, the behavioral findings should be interpreted as post-intervention between-group differences rather than direct measures of individual recovery. Future studies incorporating baseline and longitudinal assessments may provide a more precise evaluation of treatment-related behavioral recovery.

Furthermore, inflammatory mediators were assessed only at the 7-day endpoint because the present study was designed to evaluate the effects of repeated tDCS treatment over a 7-day therapeutic period [7]. Therefore, the acute temporal dynamics of TNF-α and IL-1β following TBI and their potential modulation by tDCS could not be determined. This is relevant because post-traumatic inflammatory signaling evolves over time, and some mediators may show early context-dependent protective effects before contributing to chronic injury cascades [17]. Future studies incorporating serial assessments at 6, 24, 48, and 72 h, together with the 7-day endpoint, will be necessary to characterize these temporal responses more comprehensively.

In addition, a tDCS-only group without TBI was not included; therefore, the effects of tDCS in the absence of traumatic injury could not be independently assessed. A formal a priori power calculation was not performed before study initiation. The sample size was determined based on group sizes used in our previous experimental TBI and tDCS studies; however, prospective power calculations based on predefined primary outcomes and effect-size estimates should be incorporated in future studies.

In conclusion, this study demonstrates that early anodal tDCS improves behavioral outcomes following traumatic brain injury in a young adult rat model and is associated with reduced TNF-α and IL-1β levels and changes in AChE and ChAT protein expression in the hippocampus and motor cortex. Taken together with emerging preclinical and early clinical literature, these findings support anodal tDCS as a promising but still mechanistically incompletely defined neuromodulatory strategy after TBI [35,36]. These findings provide preclinical evidence for beneficial effects of anodal tDCS following TBI; however, they do not establish restoration of cholinergic function, a definitive causal mechanism, or clinical efficacy. Further studies with longer follow-up, both sexes, direct assessment of cholinergic function, and additional mechanistic approaches are warranted to determine the durability and translational potential of these findings.

## 5. Conclusions

This study demonstrates that early anodal tDCS improves functional and molecular outcomes following young adult traumatic brain injury. Among the stimulation paradigms evaluated, anodal tDCS consistently improved locomotor activity, recognition memory, and spatial working memory while significantly attenuating trauma-induced neuroinflammation and cholinergic dysregulation in the hippocampus and motor cortex. In contrast, cathodal stimulation produced only limited therapeutic effects.

The concurrent reduction of TNF-α and IL-1β together with attenuation of trauma-induced alterations of AChE and ChAT expression suggests that anodal tDCS promotes recovery through coordinated effects on inflammatory and cholinergic pathways rather than by targeting a single mechanism. These findings provide new mechanistic evidence linking neuromodulation with improving of post-traumatic neurochemical balance in young adult rats.

Overall, our results support early anodal tDCS as a promising non-invasive therapeutic strategy for traumatic brain injury in young adults. Further studies incorporating long-term functional evaluation, electrophysiological analyses, and direct assessment of cholinergic neurotransmission are warranted to validate these findings and facilitate clinical translation.

## Data Availability

The data presented in this study are available from the corresponding author upon reasonable request.

## Supplementary Materials

The following supporting information can be downloaded at: https://www.mdpi.com/article/10.3390/biom16101428/s1, Figure S1: Original Western blot image of AChE in hippocampus; Figure S2: Original Western blot image of ChAT in hippocampus; Figure S3: Original Western blot image of AChE in motor cortex; Figure S4: Original Western blot image of ChAT in motor cortex; Figure S5: β-actin loading control.

## Abbreviations

AChE	Acetylcholinesterase
ChAT	Choline Acetyltransferase
C-tDCS	Cathodal Transcranial Direct Current Stimulation
DI	Discrimination Index
ECL	Enhanced Chemiluminescence
ELISA	Enzyme-Linked Immunosorbent Assay
HRP	Horseradish Peroxidase
IL-1β	Interleukin-1 Beta
NMDA	N-Methyl-D-Aspartate
NOR	Novel Object Recognition
OF	Open Field
PBS	Phosphate-Buffered Saline
PVDF	Polyvinylidene Fluoride
RIPA	Radioimmunoprecipitation Assay
SDS-PAGE	Sodium Dodecyl Sulfate–Polyacrylamide Gel Electrophoresis
SEM	Standard Error of the Mean
SPSS	Statistical Package for the Social Sciences
TBI	Traumatic Brain Injury
tDCS	Transcranial Direct Current Stimulation
TNF-α	Tumor Necrosis Factor-Alpha
