# Supplementary material for: Anodal Transcranial Direct Current Stimulation Reduces Neuroinflammation and Cholinergic Dysregulation After Young Adult Traumatic Brain Injury
Source: Biomolecules. 2026 Oct 1;16(10):1428. doi: 10.3390/biom16101428 (PMC13702753; doi:10.3390/biom16101428)
Supplement: Supplementary file 1 [file biomolecules-16-01428-s001.zip › biomolecules-4554284-supplementary.pdf]

Supplementary Information – Original Western Blot Images

This file contains the original uncropped Western blot images corresponding to the experiments presented in the manuscript. Each target protein (AChE, ChAT, and β-actin) was analyzed on separate gels and membranes. Therefore, the bands shown in the main figures originate from independent electrophoresis experiments rather than different regions of the same gel. No cropping or rearrangement of bands was performed. Only minimal global brightness and contrast adjustments were applied equally across the entire images to improve visualization.

AChE Hippocampus

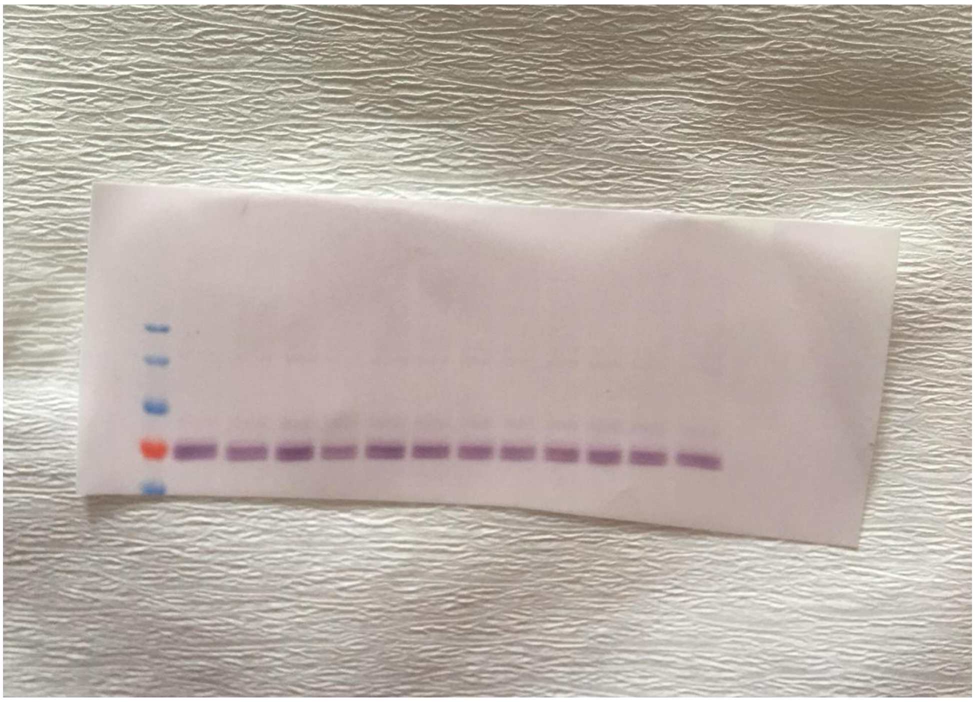

Supplementary Figure S1. Original Western blot image of AChE in hippocampus

Original and uncropped Western blot image showing acetylcholinesterase (AChE) protein expression in hippocampal tissue samples. This blot corresponds to the bands quantified in the main manuscript. Each protein was analyzed on a separate gel; therefore, this blot represents an independent electrophoresis and transfer experiment. No cropping or selective editing was performed. Only minimal and uniform brightness/contrast adjustments were applied to improve visibility.

ChaT Hippocampus

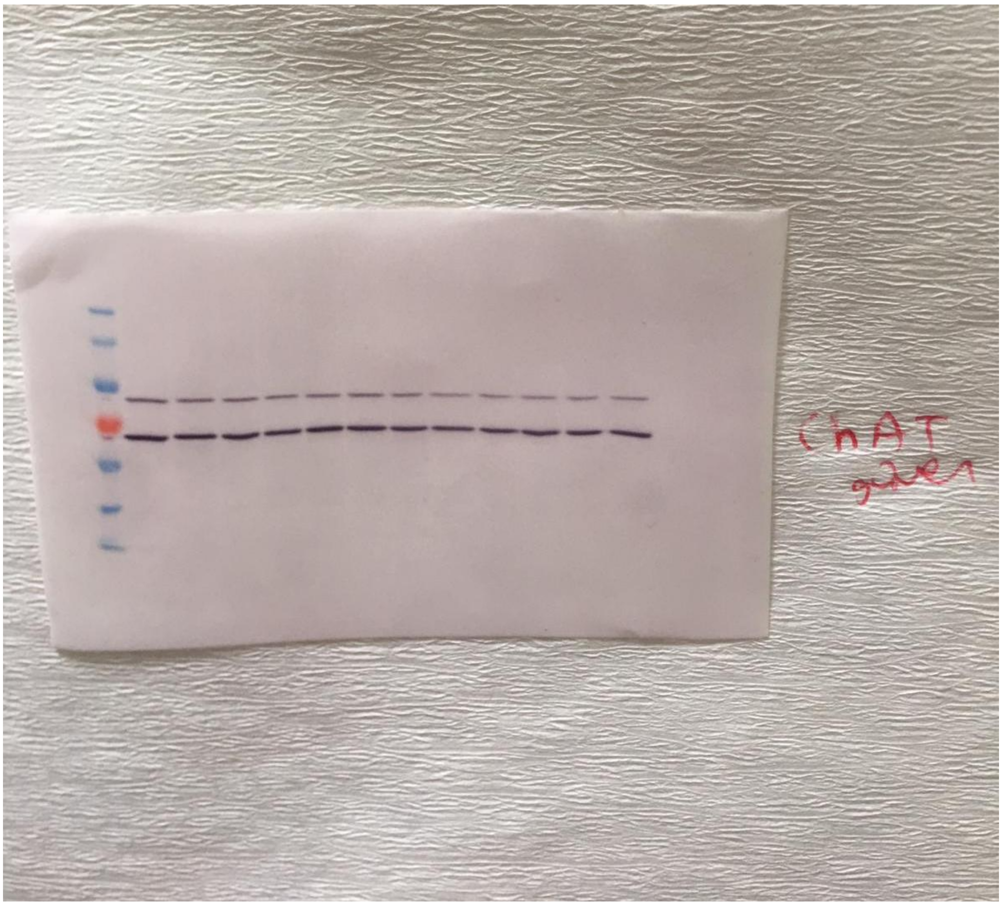

Supplementary Figure S2. Original Western blot image of ChAT in hippocampus

Original uncropped Western blot image showing choline acetyltransferase (ChAT) protein expression in hippocampal tissue samples. This blot corresponds to the quantification presented in the main manuscript. The experiment was performed on an independent gel and membrane. No bands were removed or rearranged.

AChE Motor Cortex

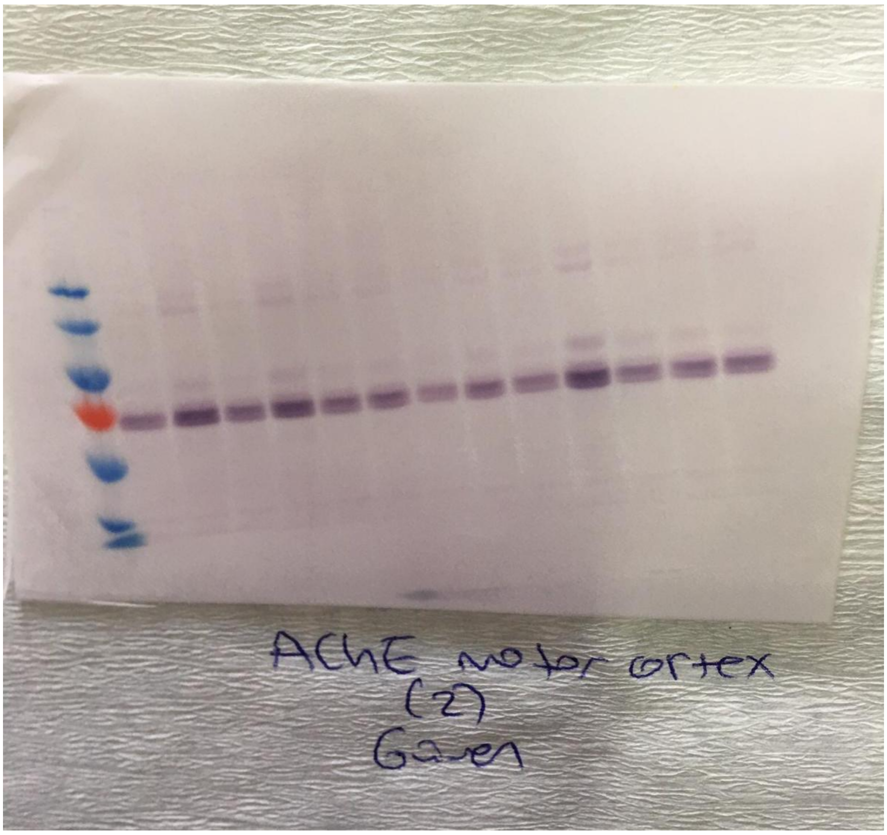

Supplementary Figure S3. Original Western blot image of AChE in motor cortex

Original uncropped Western blot image representing acetylcholinesterase (AChE) protein expression in motor cortex tissue samples. The blot is presented in its original form without cropping. Each protein was analyzed on a separate gel under identical experimental conditions.

ChaT Motor Cortex

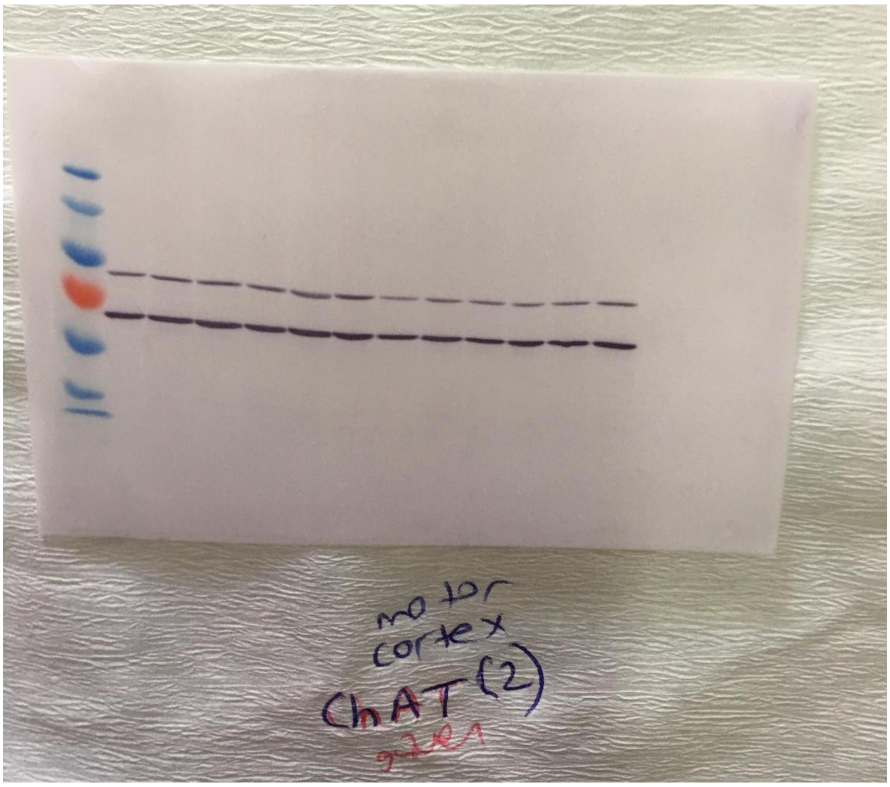

Supplementary Figure S4. Original Western blot image of ChAT in motor cortex

Original uncropped Western blot image showing choline acetyltransferase (ChAT) expression in motor cortex tissue samples. This blot represents an independent electrophoresis experiment. No cropping or selective manipulation was applied. Each protein was analyzed on a separate gel and membrane under identical experimental conditions.

B Actin

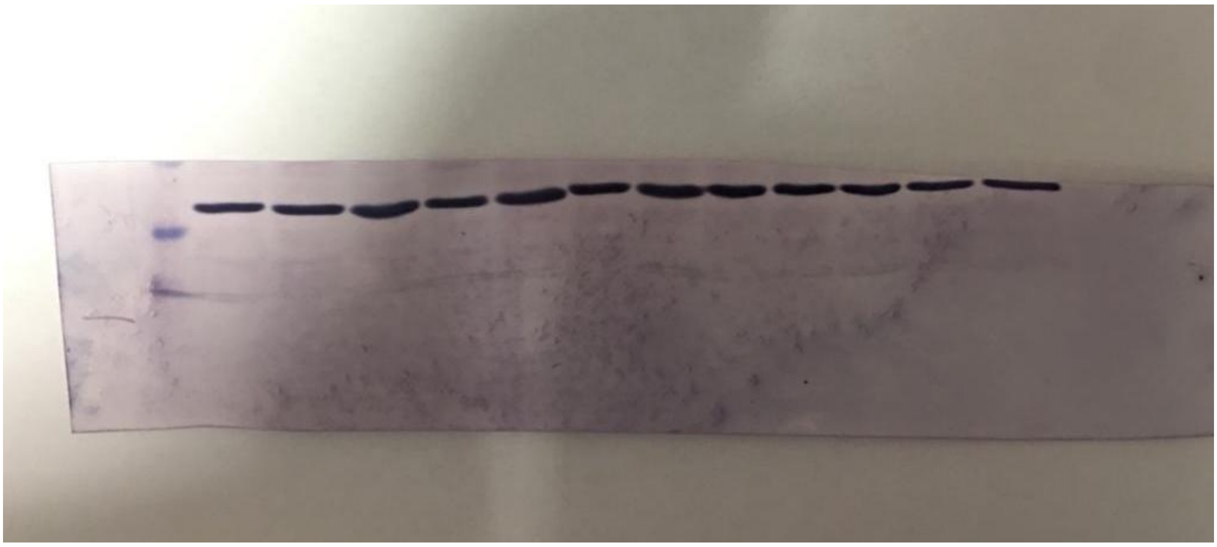

Supplementary Figure S5. β-actin loading control

Original uncropped Western blot image of β-actin used as the internal loading control for normalization of protein expression levels during densitometric analysis. β-actin was run on separate gels corresponding to the target protein experiments.
